# From Neuronal Differentiation of iPSCs to 3D Neuro-Organoids: Modelling and Therapy of Neurodegenerative Diseases

**DOI:** 10.3390/ijms19123972

**Published:** 2018-12-10

**Authors:** Matteo Bordoni, Federica Rey, Valentina Fantini, Orietta Pansarasa, Anna Maria Di Giulio, Stephana Carelli, Cristina Cereda

**Affiliations:** 1 Genomic and Post-Genomic Center, IRCCS Mondino Foundation, 27100 Pavia, Italy; matteo.bordoni@mondino.it (M.B.); orietta.pansarasa@mondino.it (O.P.); cristina.cereda@mondino.it (C.C.); 2 Laboratory of Pharmacology, Department of Health Sciences, University of Milan, via A. di Rudinì 8, 20142 Milan, Italy; federica.rey@unimi.it (F.R.); annamaria.digiulio@unimi.it (A.M.D.G.); 3 Department of Brain and Behavioural Sciences, University of Pavia, 27100 Pavia, Italy; valentina.fantini@mondino.it; 4 Laboratory of Neurobiology and Neurogenetic, Golgi-Cenci Foundation, 20081 Abbiategrasso, Italy; 5 Pediatric Clinical Research Center Fondazione Romeo ed Enrica Invernizzi, University of MilanVia Giovanni Battista Grassi, 74, 20157 Milan, Italy

**Keywords:** cell culture, iPSCs, 3D bioprinting, organoids, disease modelling, personalized medicine, regenerative medicine

## Abstract

In the last decade, the advances made into the reprogramming of somatic cells into induced pluripotent stem cells (iPSCs) led to great improvements towards their use as models of diseases. In particular, in the field of neurodegenerative diseases, iPSCs technology allowed to culture in vitro all types of patient-specific neural cells, facilitating not only the investigation of diseases’ etiopathology, but also the testing of new drugs and cell therapies, leading to the innovative concept of personalized medicine. Moreover, iPSCs can be differentiated and organized into 3D organoids, providing a tool which mimics the complexity of the brain’s architecture. Furthermore, recent developments in 3D bioprinting allowed the study of physiological cell-to-cell interactions, given by a combination of several biomaterials, scaffolds, and cells. This technology combines bio-plotter and biomaterials in which several types of cells, such as iPSCs or differentiated neurons, can be encapsulated in order to develop an innovative cellular model. IPSCs and 3D cell cultures technologies represent the first step towards the obtainment of a more reliable model, such as organoids, to facilitate neurodegenerative diseases’ investigation. The combination of iPSCs, 3D organoids and bioprinting will also allow the development of new therapeutic approaches. Indeed, on the one hand they will lead to the development of safer and patient-specific drugs testing but, also, they could be developed as cell-therapy for curing neurodegenerative diseases with a regenerative medicine approach.

## 1. Introduction

Stem cells represent a very versatile cell source, as they are able to undergo a very high number of divisions thanks to their self-renewal property and furthermore differentiate into almost all adult cell types thanks to their pluripotency characteristic [1]. In previous years, the use of stem cells research was limited, due to invasively harvesting techniques, such as through the bone marrow, adipose tissue extraction by liposuction or blood apheresis [2]. With the discovery that adult somatic cells can be reprogrammed into the so-called induced pluripotent stem cells (iPSCs) this problem has been mostly overcome, as it is now possible to generate stem cell lines with minimally invasive techniques, such as skin biopsies or, more recently, blood withdrawals [3]. These recent findings and the increased availability of iPSCs have led to an outstanding increase in the understanding of disease mechanisms and in drug screening studies. This is particularly true for neurodegenerative diseases, as it is impossible to retrieve neural cells from patients. The reprogramming of patient-derived cells also opens new opportunities for personalized medicine approaches of drug discovery. Moreover, the development of 3D organoids and the increased relevance of bioprinting techniques provided a useful tool to generate innovative cell cultures, providing 3D models in which cells can be disposed in a controlled manner and where they can grow in tissue-like structures [4]. Obviously, 3D bioprinting opened new possibilities in the field of tissue engineering, but it can be helpful also for disease modeling. In fact, the generation of a 3D scaffold that can resemble the human tissues will permit the study of neurodegenerative diseases in the so-called “brain-in-a-dish” approach. Finally, the combination of 3D bioprinting techniques with iPSCs technology will permit the development of the most realistic and reliable in vitro cell culture, allowing the study of organoids derived from patients differentiated cells, leading to a personalized medicine approach in drug testing.

## 2. Uses of iPSCs in Neurodegenerative Diseases

**Figure 1:**
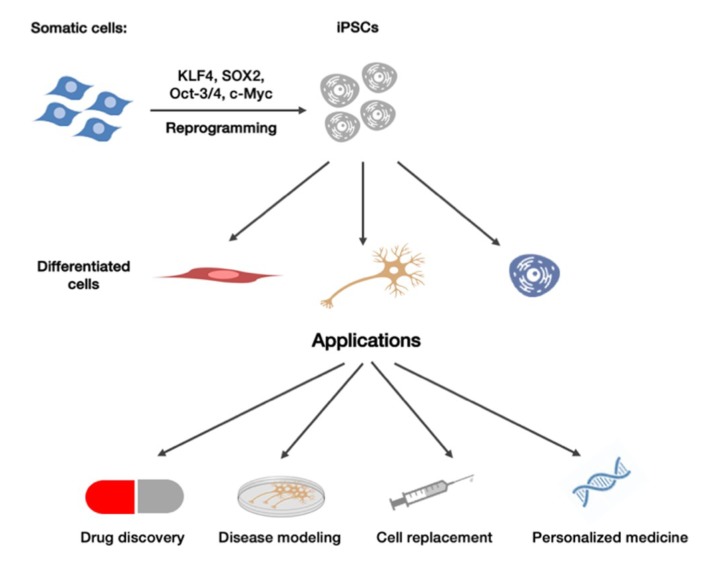
**iPSCs obtainment and potential applications.** Somatic cells can be taken from several sources and reprogrammed to iPSCs, which can be differentiated into diverse cell lines that can be used for disease modelling, drug discovery, and for cell replacement therapy.

Recent advances in the field of stem cells research have led to the development of iPSCs, which result particularly useful when related to neurodegenerative diseases. In particular, the establishment of human iPS cells has led to have an unlimited source of stem cells overcoming the ethical limits of human embryonic stem cells, which are obtained from the blastocyst, interrupting the development of a fertilized embryo [5]. Furthermore, iPSCs can be reprogrammed from any somatic cell line, allowing a less invasive retrieval and providing a new way to study diseases’ mechanisms which are patient-specific, opening to the so-called personalized medicine (Figure 1). To further advance towards this direction, it will become increasingly necessary to fully understand the pathogenic mechanisms underlying neurodegenerative diseases. Human iPSCs provide a unique opportunity to fill in some knowledge gaps, such as the genotype-phenotype association of certain neurodegenerative diseases [6]. Indeed, one of the main advantages of iPSCs is based on the ability of iPSCs to preserve many features of individual patients [7]. Moreover, neurodegenerative patients can be divided into several phenotypic categories, thus understanding disparities between such groups may lead to the finding of better diagnostic markers and to the development of fine-tuned, personalized therapies [8].

In recent years, many iPSCs’ lines have been generated from patients with neurodegenerative diseases, such as Alzheimer’s disease (AD) [9], Parkinson’s disease (PD) [10], Amyotrophic Lateral Sclerosis (ALS) [11], and Huntington’s disease (HD) [12]. 

### 2.1. IPSCs in Alzheimer’s Disease

AD is the most common form of dementia and is characterized by progressive memory-loss and declining of cognitive functions, which eventually lead to the patient’s death [13]. IPSCs-derived cells are widely used in AD’s research, and their use as in vitro models allows studies concerning both the pathogenesis of the disease and its therapy. In particular, they seem to be an appropriate model for mimicking disease mechanisms, as a higher susceptibility to amyloid beta oligomers (Aβ1-42 oligomers), typical of AD pathology [14], was found in neuronal precursors derived from iPSC of patients with a mutation in the PSEN1 gene (PSEN1-A246E mutation) [15], and in iPSCs-derived neurons of sporadic AD patients and of a patient carrying the pathogenic APP-E693Δ mutation [16]. The induction of familiar AD (fAD) mutations in neurons derived from healthy controls could also lead to in vitro models of the disease, as the expression of the PSEN1ΔE9 mutation by genome editing lead to a decrease in endocytosis and soma-to-axon transcytosis of LDL [17]. Genome editing technology could also be used for mutations’ correction, generating an isogenic control line [18]. Interestingly, iPSCs derived from iPSCs of patients with Down Syndrome, which usually have a high risk of early AD development, allowed the understanding of AD-like initial cellular hallmarks [19]. IPSCs also assume an important role when it comes to drug screening models. For example, a study found a reduction of Tau protein after treatment of iPSCs with an inhibitor of γ-secretase [20]. IPSCs-derived neurons used for drug screening need to be well differentiated, as it was seen that cells have different susceptibilities to drugs between early and late differentiation stages [21]. Finally, non-neural cells derived from iPSCs could also prove very useful in disease modeling and drug screening. Many pathological hallmarks were found aberrant in astrocytes derived from iPSCs of fAD and sporadic AD patients suggesting that astrocytic atrophy could be a plausible mechanism for early cognitive impairment [22,23]. 

### 2.2. IPSCs in Parkinson’s Disease

PD is the second most common neurodegenerative disease after AD, with a prevalence of 1% in individuals over age 60 and 4% in individuals over age 85 [24]. Usually iPSCs are differentiated into dopaminergic (DA) neurons to model PD because the disease is characterized by the loss of DA neurons of the Substantia Nigra in the midbrain. Since monogenic mutations cause an idiopathic-like disease, diverse iPSCs lines of patients with Parkin and PINK1 mutations (e.g., 2–4 exon deletion of Parkin and PINK1 Q456X) have been developed. It was seen that these cells lines present abnormalities in mitochondrial and dopamine homoeostasis, elevated α-synuclein, synaptic dysfunction, DA accumulation, microtubular stability, and axonal outgrowth, resulting in an optimal model of the disease [25,26]. Furthermore, iPSCs obtained from patients presenting the SNCA gene triplication with elevated α-synuclein showed an impairment of differentiation and maturation [27]. An electrophysiological characterization of control dopaminergic neurons derived from iPSCs was provided by Hartfield and colleagues, who confirmed that these cells have the physiological hallmarks of dopaminergic neurons previously reported only on rodent slice [28]. IPSCs have also allowed an innovative co-culture of microglial cells and cortical neurons, highlighting a unique cytokine profile impossible to obtain without iPSCs [29]. Interestingly, the pathologic phenotype was reversed in cortical neurons derived from iPSCs of patients mutated in SNCA using a small molecule found by yeast screening, opening new possibilities in drug screening and testing [30]. IPSCs also appear to be fundamental for the development of PD therapy, both for drug screening [30] and stem cell therapy [31]. Indeed, Kikuchi et al. achieved the transplantation of human iPS cell-derived dopaminergic neurons in a primate model of PD treated with 1-methyl-4-phenyl-1,2,3,6-tetrahydropyridine, with an increase in spontaneous movement of the monkeys, demonstrating for the first time that such transplantation could be clinically applicable for the treatment of PD patients [31]. 

### 2.3. iPSCs in Amyotrophic Lateral Sclerosis

ALS is the most prevalent motor neuron disease and is characterized by the progressive loss of upper and lower motor neurons (MNs), leading to muscle atrophy, paralysis, and death 2–5 years after the first diagnosis [11]. MNs derived from iPSCs are the most common neural cell type used in ALS, useful for understanding disease mechanisms. An increase in oxidative stress and in DNA damage was found in iPSC-derived C9ORF72 MNs, confirming that the reduction of oxidative stress could help to delay patients’ death [32]. Moreover, MNs derived from iPSCs with induced mutation in FUS (P525L) were used to investigate the transcriptome and microRNA, which resulted altered with implications for ALS pathogenesis [33]. The role of astrocytes was also investigated in both sporadic and mutant patients, suggesting that in ALS patients the co-culture between MNs and astrocytes causes alterations in both cell types [34,35]. Genetic engineering allowed the study of ALS-pathways, as it was found that the activation of AP1 drives neurodegeneration in genetically-corrected SOD1 mutant MNs [36]. 

IPSCs MNs also proved to be a useful model for drug screening. Small molecule compounds that regulate IGF-II expression were found to increase MN resilience [37]. Furthermore, Egawa and colleagues, which firstly generated and characterized MNs from iPSCs of TDP43 mutated patients, found some pathological hallmark, such as short neurites and abnormal insoluble TDP43. They then tested trichostatin A, spliceostatin A, garcinol, and anacardic acid and found that the last one, an inhibitor of histone deacetylase, rescued the pathogenic abnormalities [38]. 

### 2.4. iPSCs in Huntington’s Disease 

HD is characterized by loss of neurons mainly in the caudate nucleus, putamen, and the cerebral cortex with affection in a later stage of other areas, e.g., hippocampus and hypothalamus [39]. Even though the genetic cause for this disease is known, an expansion mutation of the trinucleotide (CAG) repeat in the HTT (IT15) gene [40], the mechanisms through which mutant HTT results in the degeneration of selective neurons population are still unclear. Thus, studies on HD models are needed in order to discover treatments. 

Neurons differentiated from iPSCs of patients helped to understand the role of mutant HTT gene and the mechanisms that lead to the pathology. For example, early molecular changes in intracellular signaling, expression of oxidative stress proteins, and the p53 pathway were reported in both iPSCs and in iPSCs-derived neurons [41]. Another study reported changes in neuronal development and adult neurogenesis, exploiting the iPSCs capacity to model embryonal development [42]. The possibility to differentiate iPSCs into neurons opened the possibility to discover new therapeutic targets, such as pre-mRNA trans-splicing modules [43]. Finally, the role of glial cells was investigated in several studies, such as the one carried out by Hsiao and colleagues, which reported that HD astrocytes provide less pericyte coverage, reducing their number and promoting angiogenesis [44]. 

## 3. From iPSCs to “Brain on a Dish”: Role of Brain Organoids in Neurodegenerative Diseases

**Figure 2:**
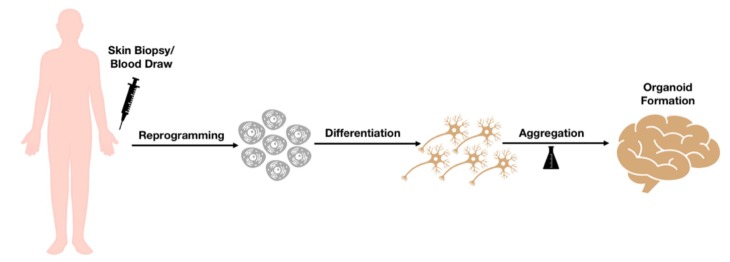
**Workflow of human organoids obtainment.** Patients’ derived cells (fibroblasts or peripheral blood mononuclear cells) are reprogrammed to iPSCs, which are then differentiated to neural precursors. These are directed towards the formation of aggregates (typically with the use of spinning bioreactors) which are then organized into a cerebral organoid structure.

In the last few years, advances have been made to move on from a 2D to a 3D approach in order to gain further insights into the cytoarchitecture of the brain and into the disease mechanisms that may take place in the central nervous system. Both methods present with advantages and disadvantages: while 2D models with directed monolayer differentiation may provide an easier substrate for imaging assays and morphological studies such as dendrite complexity, 3D cultures can result in a large diversity of cell types and allow the study of cell-cell interactions between different populations [45,46]. 3D human brain cultures are usually obtained with the differentiation of iPSCs into either neural cell aggregates or into more complex brain organoids. Neural cell aggregates are usually generated from human neural progenitors cultured in a 3D suspension, whilst brain organoids are mainly derived with serum-free floating culture of embryoid body-like aggregates [47] (Figure 2). Lancaster et al. used a derivation of this last method to obtain a novel class of organoids known as “mini-brains”, containing various discrete but interdependent brain regions [48]. Brain organoids actually have certain limits, because not every aspect of brain diseases can be represented by such models, but future studies will allow to advance brain organoid techniques for modeling neurodegenerative diseases, thus elucidating novel aspects of disease pathogenesis [49].

### 3.1. Applications of Brain Organoids

**Figure 3:**
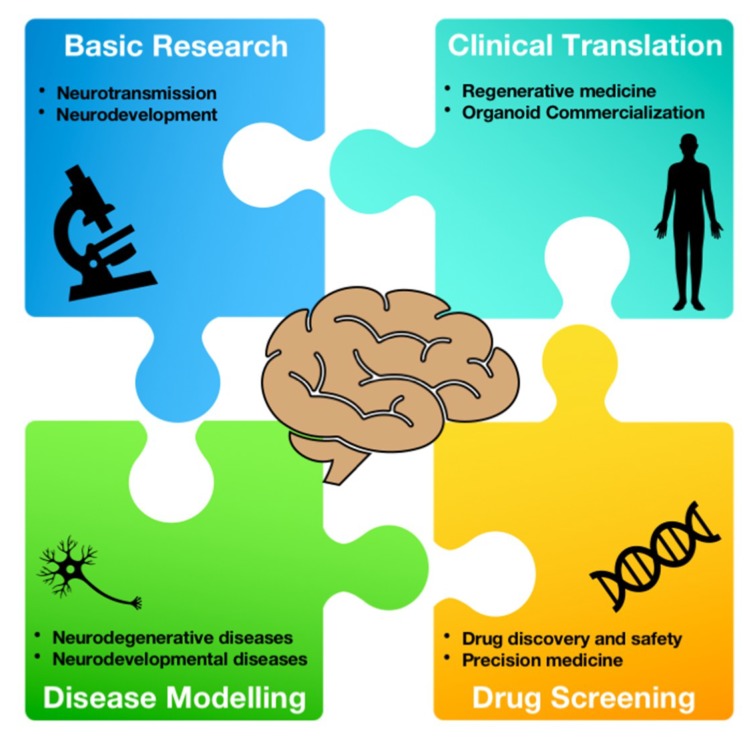
**Potential applications for organoid research.** The obtainment of human-derived organoids allows for a comprehensive understanding of neurological disorders. Their use in basic research allows for studies investigating the brain’s structure and connections. Moreover, their role in disease modelling will allow for the investigation of potential alterations in cerebral organoids derived from patients with neurodegenerative or neurodevelopmental diseases. They will provide a useful platform for drug screening, and will lead the way to precision medicine approaches, allowing for a more directed and safe clinical translation.

Even more than with iPSCs, brain organoids will help us gain insights into disease mechanisms of neuronal disorders and indeed will provide a useful tool for drug screening and new therapeutic approaches. The first and main application for these 3D cultures is the study of neurodevelopmental disorders, which has found great interest and made major advances with the rise of these cultures. Brain organoids derived from iPSCs of patients were fundamental in identifying developmental abnormalities and even new target genes in complex neurodevelopmental diseases, such as autism spectrum disorders [50] and microcephaly [48]. Furthermore, they resulted useful even in modeling the effects of viruses such as the Zika virus on brain development, providing a useful tool for drug screening and potential therapeutic strategies [51]. More recently, combined approaches of 3D modeling and genetically engineering allowed the development of cerebral organoids to model brain tumors, providing a platform for investigating the tumor biology of such aggressive cancers and new preclinical model for drug screening [52]. Lastly, nowadays brain organoids are gaining a more prominent role also for the study of neurodegenerative diseases (Figure 3). 

#### Brain Organoids in Neurodegenerative Diseases

When considering brain organoids and neurodegenerative diseases, it must be taken into consideration the late-onset of these diseases, which usually occur when the brain is fully formed, resulting in a need to create a more “aged brain”. Although not every aspect of the aged brain can be modeled by such organoids, earlier events in disease progression may share molecular and cellular mechanisms that usually appear at later stages in neurodegenerative diseases [49]. Indeed, cerebral organoids have still proven to be a useful tool for the study and drug screening of neurodegenerative diseases such as AD, HD, and PD [47,53]. Raja et al. obtained a self-organizing 3D human formation from iPSCs derived from AD patients with a duplication in the APP gene. These structures presented with the main hallmarks of the disease, Aβ aggregation and Tau phosphorylation, and they proved useful for the screening of b- and y-secretase inhibitors, which resulted in a decrease of the aggregates formation [54]. For Huntington’s disease, 3D organoids allowed the study of the effect of the huntingtin mutation on aspects of neuronal development such as abnormal cell organization and precocious acquisition of mature neuronal identities. Indeed, also in this case, pharmacological intervention rescued the correct neuronal differentiation [55]. Regarding Parkinson’s Disease, only one organoid model has been developed, containing the G2019S LRRK2 mutation, which highlighted the synaptic dysfunction pathway as the most altered one in patients derived organoids. Intriguingly, this work also derived gastrointestinal organoids from the iPSCs of the same class of patients, highlighting differences in gene expression of intestinal cells of patients as opposed to controls [56]. 

### 3.2. Future Advances for 3D Cultures: Importance of Biomaterials

There are some limitations with 3D brain organoids, the main one being the limited reproducibility of these cultures. This is particularly true for 3D organoids which aim to mimic later stages of development, as the proportion of cell types which can generate from iPSCs may diverge resulting in non-identical models [45]. To overcome this, fundamental will be the use of biomaterials, which will allow the scaffolding of brain cultures, creating a niche-like effect and confining progenitors in a non-uniform environment which can resemble the actual situation present in during neural development. Substrates like the hydrogel are first of all highly programmable and can, furthermore, be manipulated both in their pore size and physical properties, creating more complex structures which can “aid” the neural differentiation of progenitors [57].

## 4. Recent Developments in 3D Bioprinting

**Figure 4:**
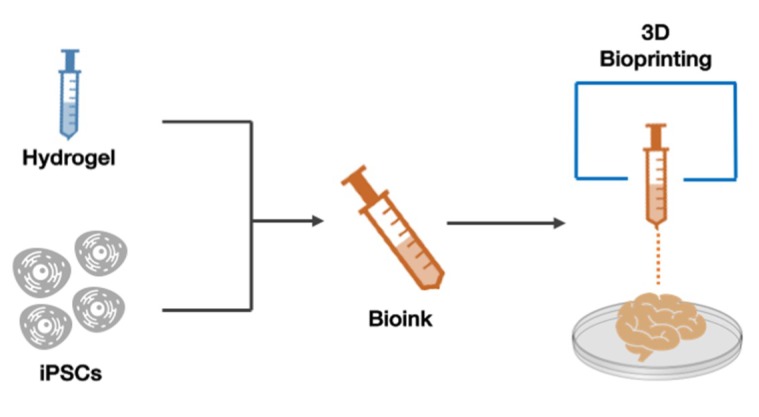
**3D bioprinting technology.** Hydrogel and iPSCs are combined to obtain a bioink, which can then be used to design and print three-dimensional structures, such as organoids.

3D bioprinting is an emerging technology, used for the manufacture and the generation of artificial tissues and organs [58], adding new approaches to tissue engineering (TE) and regenerative medicine, such as the manufacture of scaffolds to support cells, as well as in situ deposition of cell suspensions [59]. Bioprinting technology has allowed to overcome several limits, such as the control of in vitro 3D biological structures and cellular distribution [60]. Bioprinting, through the use of hardware and software, has been used in particular for the design of three-dimensional structures, allowing the creation of “organoids” for biological and pharmacological studies, and the repair and replacement of human tissues (Figure 4).

One of the fundamental elements that characterizes the bioprinting process is the development of biomaterials, which must have specific characteristics: biocompatibility, printability, and the ability to maintain a three-dimensional structure once printed and kept in culture [58]. The main feature of the hydrogel, the main biocompatible material used as a three-dimensional support for cell growth, is the ability to be extremely hydrophilic, making it an excellent candidate in terms of biocompatibility for its use in bioprinting. It was initially used in TE because it was able to simulate the extracellular matrix, guaranteeing cell growth and communication [61]. Several cell types associated with different biomaterials that compose the bioink have already been used in several research areas, where cellular viability and motility have been demonstrated, as well as a spatial organization similar to in vivo tissue [62]. Researchers tend to create a combination of biomaterials for each cell type, and with well-defined printability specifications, so as to make the process as standardized and reproducible as possible, despite being a very open field and full of new developments. New generation bioinks are now able to maintain each of these characteristics, thus improving the success in terms of bioprinting [63]. 

### 3D Bioprinting and Neurodegeneration

In the last decade, the possibility of replacing dead cells in neurodegenerative processes paved the way for a more intense and accurate study of stem cells and their potential ability to replace damaged tissue [64]. It was also thought to exploit the ability of stem cells to secrete cytokines and growth factors, offering benefits such as anti-inflammatory effects, protection of neural cells, and endogenous recovery systems. Transplanting these cells into damaged sites presents various problems such as low cell survival and limited engraftment [65]. To minimize these problems, it was decided to use three-dimensional scaffold printing that mimic the complexity both from the biological and functional point of view of the tissue to be replaced [66].

The manufacture of three-dimensional prefabricated scaffolds has already given positive results in the treatment and repair of spinal and nerve damage, but with a great limitation in terms of control of the external shape of the scaffold and of its internal architecture [67,68]. These problems have been overcome with 3D bioprinting, which leaves the operator complete freedom regarding the shape, the material, and its internal architecture. The recent developments in the field of 3D bioprinting are mostly focused on regenerative medicine, to respond to the growing demand for tissues and organs for transplants, arriving only later to the application of this technology to basic scientific research. Until now only a few studies have focused on using 3D printing applied to the creation of neural tissue, compared to other widely studied tissues such as skin, bones, heart tissue, and cartilaginous structures [69]. For neural tissue Lozano and colleagues developed a novel printing technique for engineering 3D brain structures to model neurodegenerative diseases. They encapsulated primary cortical neural cells in gellan gum modified with RGD peptides. They found that cortical neurons remained viable throughout the printed construct and exhibited differentiated neurons morphology [70]. Moreover, Kuzmenko and colleagues proposed nanofibrillated cellulose (NFC) functionalized with carbon nanotubes (CNTs) as a supporting material for neural cells. They hypothesized that NFC provides a surface roughness that enhances attachment of cells, while the functionalization with CNTs allows significant improvement of cellulose conductivity (about 10^5^ times increase), favoring communications between cells and generation of a neural network [71]. In our experience, we tested hydrogels with several concentrations of sodium alginate and gelatin [72]. Specifically, we found that the best bioink for neural cells in terms of proliferation, viability, and structural support is the one composed of 6% of sodium alginate and 4% gelatin. Our data suggest that the bioink we developed improves cellular proliferation, compared with 2D standard cell cultures, obtaining in less time a larger number of cells with an increased viability. The three-dimensionality, fundamental for cell-to-cell communication, is also maintained. The printing process was standardized in term of print pressure, temperature, and speed, to print our hydrogel with Cellink INKREDIBLE+ bioplotter, with a good repeatability and a robust preparation protocol. Indeed, it is necessary to establish a repeatable 3D bioprinting protocol, in order to standardize cellular differentiation and factors deposition, to facilitate cell organization, and also because matrigel-based cell cultures do not guarantee a defined 3D spatial assembling. In the future, we will test electrical communication between neural cells in printed construct, in order to verify the network operation, using differentiated neural stem cells, and to create a detailed and realistic neural tissue [72].

Recently, researchers also think that the nervous tissue printed in 3D may be used for the neural regeneration, a with very large potential in the field of neurodegeneration to replace degenerated neural tissue [73,74]. Obviously, 3D bioprinting is a relatively new application to the tissue engineering field, opening the possibility to generate a remarkable biomimicry, which could replace the current gold standard of autografts [75,76]. 3D bioprinting technologies allow several adjustments to the shape, porosity, and size of the 3D bioprinted scaffolds, which could make them more suitable in the repair of the lesion site in neurodegenerative diseases with respect to simply delivering the cells using injectable hydrogels. Even so, but several challenges remain, such as the development of bioinks. Probably, in the future, it will be necessary to develop new biomaterials and increase the precision of bioplotter, allowing the generation of accurate 3D structures [77].

The creation of nerve tissue by bioprinting is also used for pharmacological studies, for toxicological screening, and for basic research. It is necessary to underline how this field is still in its infancy, how it is necessary to validate this model for the applications described up to now, to be sure that the model completely recapitulates the pathophysiology that we want to investigate with this tool [66] in particular with regard to neurodegenerative diseases.

## 5. Conclusions

In the last decade, two groundbreaking discoveries, i.e., somatic cells reprogramming into iPSCs and 3D bioprinting, changed the way of modeling diseases, in particular for those pathologies difficult to study in a simple cell culture, such as neurodegenerative diseases. The first permitted to obtain neural cell cultures in few months starting from adult somatic cells, like fibroblasts and peripheral blood mononuclear cells, while 3D bioprinting consists in the print of hydrogel and cells, to generate models that imitate tissue characteristics. While iPSCs are differentiated into neurons and, furthermore, into brain organoids in many papers for disease modeling, 3D bioprinting is actually used for few tissues, like cartilage, bone, and heart. Neural 3D cell cultures are still in development, there are no target bioinks and the studies that combine neuronal cells and 3D bioprinting is more complicated with respect to other tissues because of the fragility of such cells. Despite this hurdle, the possibility to create an in vitro neural tissue would open many fields of research that are unreachable today; first of all, the opportunity to study the 3D spatial connection between different neuronal populations, and how they communicate with each other. In combination with iPSCs and organoids technology, we can create a physiological model to understand physiological and pathological mechanisms, to better understand mechanisms underlying neurodegenerative diseases. 

Finally, the combination between 3D bioprinting and organoids will open new possibilities in many fields: drug screening, replacing expensive in vivo experiments, and overcoming animal models’ issues, but also personalized medicine thanks to the use of cells derived from patients. More intriguingly, the generation of a 3D neural tissue composed of patient’s cells will allow so-called neuro-regeneration, opening the possibility to replace a degenerated tissue.

## Abbreviations

IPSCs	Induced pluripotent stem cells
AD	Alzheimer’s disease
PD	Parkinson’s disease
ALS	Amyotrophic lateral sclerosis
HD	Huntington’s disease
fAD	familiar AD
DA	Dopaminergic
MNs	Motor neurons
TE	Tissue engineering
